# Urine Albumin‐to‐Creatinine Ratio as an Indicator of Brain Activity Changes in Chronic Kidney Disease: A Resting‐State fMRI Study

**DOI:** 10.1002/brb3.70106

**Published:** 2024-10-17

**Authors:** Yangjie Yu, Jun‐Peng Zhang, Zhen Wang, Juan Li, Xu‐Yun Hua, Junjie Pan, Rui Dong

**Affiliations:** ^1^ Department of Cardiology, Huashan Hospital Fudan University Shanghai China; ^2^ School of Rehabilitation Science Shanghai University of Traditional Chinese Medicine Shanghai China; ^3^ Department of Radiology Changhai Hospital Shanghai China; ^4^ Department of Nephrology Changhai Hospital Shanghai China; ^5^ Engineering Research Center of Traditional Chinese Medicine Intelligent Rehabilitation Ministry of Education Shanghai China; ^6^ Department of Traumatology and Orthopedics Shuguang Hospital Affiliated to Shanghai University of Traditional Chinese Medicine Shanghai China

**Keywords:** amplitude of low‐frequency fluctuation, chronic kidney disease, regional homogeneity, resting‐state fMRI

## Abstract

**Objective:**

Chronic kidney disease (CKD) is increasingly recognized as a risk factor for alterations in brain function. However, detecting early‐stage symptoms and structural changes remains challenging, potentially leading to delayed treatment. In our study, we aimed to investigate spontaneous brain activity changes in CKD patients using resting‐state functional magnetic resonance imaging (fMRI). Additionally, we explored the correlation between common biomarkers reflecting CKD severity and brain activity.

**Methods:**

We recruited a cohort of 22 non–dialysis‐dependent CKD patients and 22 controls for resting‐state fMRI scans. Amplitude of low‐frequency fluctuations (ALFFs) and regional homogeneity (ReHo) were calculated to evaluate brain activity. Regression analysis was conducted to explore the correlations between biomarkers reflecting the severity of CKD and brain activity.

**Results:**

CKD patients exhibited reduced z‐scored ALFF (zALFF) and mean ALFF (mALFF) in the bilateral putamen, right caudate nucleus, left anterior cingulate, and right precuneus. Changes in bilateral putamen were also found in smCohe‐ReHo and szCohe‐ReHo analyses. Urine albumin‐to‐creatinine ratio (UACR), urine protein‐to‐creatinine ratio (UPCR), and serum albumin levels were associated with attenuated putamen activity.

**Conclusion:**

Non–dialysis‐dependent CKD patients had changes in zALFF, mALFF, smCohe‐ReHo, and szCohe‐ReHo values in specific brain regions, especially bilateral putamen. UACR, UPCR, and serum albumin levels are associated with putamen activity attenuation in rs‐fMRI.

## Graphical abstract

A cohort comprising 22 non–dialysis‐dependent chronic kidney disease (CKD) patients and 22 healthy controls underwent resting‐state functional magnetic resonance imaging (fMRI) scans, coupled with biochemistry examinations. The amplitude of low‐frequency fluctuations (ALFFs) and regional homogeneity (ReHo) were calculated. Notably, alterations within the bilateral putamen regions were identified through both ALFF and ReHo analyses. Furthermore, linear regression analyses revealed that urine albumin‐to‐creatinine ratio (UACR), urine protein‐to‐creatinine ratio (UPCR), as well as serum albumin levels were significantly associated with putamen activity.

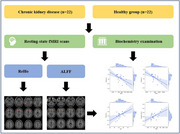

## 1 Introduction

Chronic kidney disease (CKD) is diagnosed when the abnormal structure or function of the kidney is presented for over 3 months (Roumeliotis, Mallamaci, and Zoccali 2020; Skalsky et al. 2022). It is a widespread health condition that impacts 9.6% of the world's population (Bikbov et al. 2020; Murton et al. 2021). Since 2013, epidemiologic data have indicated that individuals at all stages of CKD have a higher risk of developing cognitive disorders and dementia, and that may lead to lower quality of life (Bugnicourt et al. 2013; Hamed 2019). The maximal exercise capacity has been reduced substantially among the patients with CKD (Leikis et al. 2006; McManus et al. 2007; Odden, Whooley, and Shlipak 2004). CKD has also been found accompanied by motor control alterations, such as postural instability, hand tremor, and reduced gait speed (Lee et al. 2015; Tran et al. 2019). Besides, visuospatial dysfunction was found in CKD patients in several previous studies (Jawa et al. 2022; Zhang et al. 2018). The most recent review also suggests that CKD leads to central nervous system complications (Hamed 2019; Kim, Kim, and Gil 2022). However, the dysfunctions mentioned above and brain structure changes are more common in the end stage of the CKD population (Song et al., 2023a, 2023b, 2023a, 2023b). Effective intervention is already difficult when these changes are discovered. Therefore, it is crucial to identify and screen people who are prone to brain function alteration earlier.

Resting‐state functional magnetic resonance imaging (rs‐fMRI) is a novel non‐invasive imaging approach that may aid in the exploration of disparities between CKD patients and the normal populace before structure changes occur (Li et al. 2017, 2020; Yang et al. 2024). Rs‐fMRI examines the blood oxygen level–dependent (BOLD) signal resulting from the entire brain's spontaneous fluctuations. During the examination, patients are not required to engage in cognitive activities. After examination, the amplitude of low‐frequency fluctuation (ALFF) can be utilized to study spontaneous brain activity based on rs‐fMRI data (Hillman 2014). Regional homogeneity (ReHo) analysis reflects the synchronization of whole‐brain voxels in localized regions of brain activity state (Liu et al. 2010). Its measurements are positively correlated with the coherence and centrality of regional brain activity. There have been several fMRI studies targeting patients undergoing dialysis in the end stage of CKD. An rs‐fMRI study using the ALFF and functional connectivity algorithm suggested that in hemodialysis patients, the brain areas with abnormal spontaneous brain activity and functional connectivity are mainly located in the default mode network (DMN) regions (Su et al. 2021). Another study found that hemodialysis patients had aberrant ALFF in the DMN regions, particularly in the precuneus, which may be correlated with neuropathological mechanisms involved in both CKD and hemodialysis (Chen et al. 2020). Patients in end‐stage CKD undergoing peritoneal dialysis also have impaired brain networks (C. Zhang et al. 2023), even among those with preserved cognitive function (Chang et al. 2021). However, the spontaneous brain activity changes in these patients might be strongly affected by dialysis (Peng et al. 2021). Brain activity changes in non‐dialysis‐dependent CKD patients were less investigated.

fMRI provides valuable insights into brain function, but its widespread clinical use remains challenging due to time constraints and limited availability in many hospitals. In the context of CKD, understanding brain abnormalities is essential. However, it is equally crucial to explore potential associations between these brain regions and established biomarkers. In this study, we identified abnormal brain areas in CKD patients using rs‐fMRI. Subsequently, we investigated whether changes in these brain regions correlate with clinical biomarkers, including estimated glomerular filtration rate (eGFR), B‐type natriuretic peptide (BNP), blood urea nitrogen (BUN), serum creatinine, serum albumin, and urine protein. The identification of kidney‐specific biomarkers associated with brain function is pivotal for developing targeted interventions and therapies to mitigate brain function decline and enhance the quality of life for CKD patients.

## 2 Methods

### 2.1 Participants

A total of 22 CKD patients (including 13 males; aged 56.32 ± 12.46 years) and 22 controls (including 13 males; aged 55.68 ± 13.59 years) were recruited from August 1, 2020 to May 31, 2023. All study procedures followed the Ethical Principles for Medical Research Involving Human Subjects (WMA Declaration of Helsinki). This controlled study of CKD was ratified by the ethics committee of Shanghai Changhai Hospital, Naval Medical University (No. CHEC2022‐159).

The inclusion criteria are as follows: (1) right‐handed; (2) age ≥ 30 years and < 80 years; (3) glomerular filtration rate < 60 mL/min/1.72 m^2^ in CKD participants; (4) course of CKD > 1 year; (5) could finish MR examination; (6) agreed to provide written informed consent. The exclusion criteria were as follows: (1) active glomerulonephritis or undergoing immunoinhibiting therapy; (2) undergoing renal biopsy or other treatments; (3) undergoing renal replacement therapy (hemodialysis or peritoneal dialysis); (4) cancer, infection, or fever; (5) diagnosed with central nervous system or neuropsychiatric diseases, such as cerebral infarction, depression; (6) diagnosed with psychiatric or neurologic diseases or illnesses impairing cognitive function; (7) functional insufficiency of the heart, lung, and liver; (8) taken medication that may affect cognitive function; (9) MRI contraindications.

### 2.2 Clinical Evaluations and Laboratory Examination

All patients with CKD underwent blood biochemistry examination before MRI scanning. Laboratory indicators included estimated glomerular filtration rate (mL/min/1.73 m^2^), hemoglobin A1c (%), B‐type natriuretic peptide (pg/mL), blood urea nitrogen (mmol/L), serum creatinine (µmol/L), carbon dioxide combining power (mmol/L), albumin (g/L), urine protein (g), urine albumin‐to‐creatinine ratio (UACR) (mg/g), urine protein‐to‐creatinine ratio (UPCR) (mg/g), C‐reactive protein (mg/L), erythrocyte sedimentation rate (mm/H), parathyroid hormone (pg/mL), calcium (mmol/L), phosphorus (mmol/L), lymphocyte (%), and hemoglobin (g/L).

### 2.3 MRI Data Acquisition and Pre‐Processing

All the imaging data were scanned from the same Magnetom Trio A 3T MR Scanner (Siemens AG, Erlangen, Germany) at Changhai Hospital. The heads of participants were fixed by the frame to prevent head motion. And they were informed to keep a rest and awake state before scanning. The resting state scanning sequence was a gradient‐echo echo‐planar imaging (GRE‐EPI) sequence, the detailed parameters are as follows: field of view = 240 × 240 mm^2^, matrix size = 64 × 64, slice number = 31, spacing between slices = 4.5, and the scanning order was interleaved, slice thickness = 3.6 mm, acquisition voxel size = 3.5 × 3.5 × 4.5 mm^3^; flip angle = 90°, repetition time/echo time = 2000/30 ms, and there were 240 repetitions.

The Statistical Parametric Mapping 12 (SPM 12) toolbox (Welcome Department of Cognitive Neurology, University College, London, UK; http://fil.ion.ucl.ac.uk.spm/) and the Resting‐State fMRI Data Analysis Toolkit plus (Jia et al. 2019) (http://www.restfmri.net), which are based on Matlab (2013b) (MathWorks https://www.mathworks.com/products/matlab.html), were adopted to perform the pre‐processing steps (Jia et al. 2019). Including removing the first 10 repetitions, slice timing, head movement corrections, normalization by the EPI template of the Montreal Neurological Institute space (resampled into voxel‐wise 3.0 × 3.0 × 3.0 mm^3^), spatial smoothing with a 6‐mm full‐width at half‐maximum Gaussian kernel, temporal detrending, and regressing out of covariates. After checking the data, one patient was excluded by the excessive head motion (> 2° and 2 mm) (W. K. Li et al. 2022). The data of the remaining participants were analyzed using the following steps.

### 2.4 Amplitude of Low‐Frequency Fluctuation Calculation

ALFF is usually considered that the BOLD signals detected by fMRI reflect changes in neuronal activity (Hillman 2014; Rogachov et al. 2016). The ALFF reveals the amount of fluctuation that occurs in a given brain region. To study the relationship between altered plasticity and CKD, we calculated and standardized the ALFF values by dividing the ALFF value of each voxel by the global mean values. We then transformed the ALFF values to z‐scored ALFF (zALFF) values using a Fisher *r*‐to‐*z* transformation, which allowed us to compare them among the groups (Yang et al. 2007). We also calculated mean ALFF (mALFF) (Ge et al. 2022).

### 2.5 Regional Homogeneity Calculation

ReHo is a method that assumes that a voxel in a brain area correlates highly with its 26 neighboring voxels in the time series. We calculated the coherence‐based regional homogeneity (Cohe‐ReHo) as follows (Liu et al. 2010). First, we utilized Welch's modified periodogram averaging methods to estimate the power spectra and a cross spectrum in a given cluster. Second, we calculated the coherence across the low‐frequency (0.01–0.08Hz) band with the band‐averaged estimates of the cross spectrum and the power spectrum. Third, we calculated the Cohe‐ReHo within the given cluster, and the averaged coherence coefficient of the cluster was assigned to its center voxel to represent the Cohe‐ReHo of the cluster. Thus, we obtained an individual Cohe‐ReHo map in a voxel‐wise way. We calculated smCohe‐ReHo as follows. The ReHo value of each voxel was divided by the mean value of the whole brain signal amplitude and then spatially smoothed using a 6‐mm isotropic FWHM Gaussian kernel to generate the ReHo maps for statistical analysis. A Fisher *r*‐to‐*z* transformation and spatially smooth were also performed to get szCohe‐ReHo (Agarwal, Sair, and Pillai 2017; M. Li et al. 2022).

### 2.6 Statistical Analyses

We used SPSS Statistics (version 20.0) to statistically analyze the clinical data and laboratory indicators. Gender was expressed as a number and compared using the chi‐square test. Age and BMI were expressed as mean ± standard deviation and compared using *t*‐test. Laboratory indicators of CKD patients were expressed as mean ± standard deviation. The values of ALFF and ReHo were calculated by two‐sample *t*‐tests. The results were corrected for multiple comparisons using a combined threshold of a single voxel (*p* < 0.05) with false discovery rates (FDR) correction. We chose the ROIs with statistical significance in the zALFF analysis listed in Table 2 and performed linear regression analysis for all laboratory indicators. We plotted indicators with *R* ^2^ > 0.3 and *p* < 0.01. All correlation analyses were performed in Python and used the ‘pandas’, ‘statsmodels.api’, ‘matplotlib.pyplot’, and ‘seaborn’.

## 3 Results

### 3.1 Demographic and Clinical Characteristics of CKD Patients

We included 22 patients with CKD and 22 healthy controls in our study. Baseline information for two groups is given in Table 1. Both groups were well‐matched in terms of gender and age. No statistically significant difference in body mass index (BMI) was observed between the CKD patients and healthy controls. Laboratory data were collected exclusively from the CKD group (see Table 1).

**TABLE 1:** Demographic characteristics of the total sample.

	Healthy control (*n* = 22)	CKD group (*n* = 22)	*t*/*χ* ^2^ value	*p*‐value
Male	13(59%)	13(59%)	0	1.000
Age	55.68 ± 13.59	56.32 ± 12.46	−0.162	0.872
BMI	24.42 ± 2.22	25.77 ± 3.510	−1.527	0.134
eGFR (mL/min/1.73 m^2^)	—	17.33 ± 10.39	—	—
HbA1C (%)	—	6.78 ± 1.30	—	—
BNP (pg/mL)	—	241.10 ± 256.50	—	—
BUN (mmol/L)	—	18.78 ± 8.10	—	—
SCr (µmol/L)	—	426.95 ± 297.07	—	—
CO_2_CP (mmol/L)	—	20.82 ± 2.06	—	—
ALB (g/L)	—	33.55 ± 6.57	—	—
UP (g)	—	4686.78 ± 2923.26	—	—
UACR (mg/g)	—	3511.6 ± 2448.65	—	—
UPCR (mg/g)	—	4887.84 ± 3439.45	—	—
CRP (mg/L)	—	5.19 ± 9.14	—	—
ESR (mm/H)	—	36.87 ± 26.53	—	—
PTH (pg/mL)	—	128.85 ± 73.44	—	—
Ca2+ (mmol/L)	—	2.09 ± 0.21	—	—
P (mmol/L)	—	1.63 ± 0.45	—	—
WBC (*10^9^/L)	—	6.69 ± 2.30	—	—
LYM%	—	23.86 ± 5.33	—	—
HGB (g/L)	—	105.09 ± 24.44	—	—

Abbreviations: ALB: albumin; BMI: body mass index; BNP: B‐type natriuretic peptide; BUN: blood urea nitrogen; Ca^2+^: calcium; CO_2_CP: carbon dioxide combining power; CRP: C‐reactive protein; eGFR: estimated glomerular filtration rate; ESR: erythrocyte sedimentation Rate; HbA1c: hemoglobin A1c; HGB: hemoglobin; LYM: lymphocyte; P: phosphorus; PTH: Parathyroid hormone; SCr: serum creatinine; UACR: urine albumin‐to‐creatinine ratio; UP: urine protein; UPCR: urinary protein‐to‐creatinine ratio; WBC: white blood cell.

### 3.2 Comparison of Amplitude of Low‐Frequency Fluctuations Between CKD and Healthy Control Groups

**FIGURE 1:**
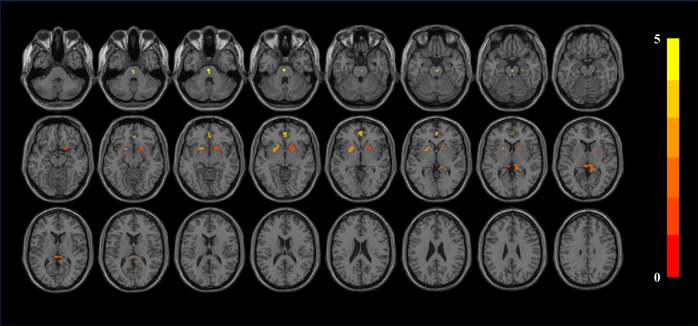
zALFF results. Difference between HC and CKD groups. The results were corrected by FDR, where *p *< 0.05 and cluster size over 20.

**FIGURE 2:**
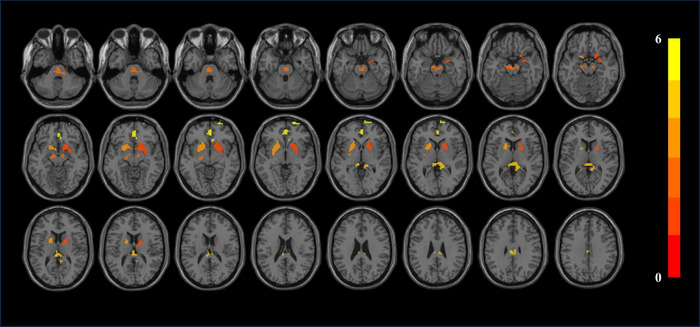
mALFF results. Difference between HC and CKD groups. The results were corrected by FDR, where *p *< 0.05 and cluster size over 20.

Patients with CKD exhibit decreased zALFF in several brain regions, including the bilateral putamen, right caudate nucleus, left anterior cingulate and paracingulate gyri, and right precuneus. These alterations are statistically significant (*p *< 0.05) and involve clusters with a size greater than 30 voxels (see Figure 1 and Table 2). Similarly, patients with CKD demonstrate decreased mALFF in bilateral putamen, bilateral caudate nucleus, left anterior cingulate, and right precuneus. These mALFF changes are also statistically significant (*p *< 0.05) and occur within clusters exceeding 30 voxels (see Figure 2 and Table 3).

**TABLE 2:** Brain regions with significant differences in zALFF between CKD and HC.

				Peak MNI Coordinates		
Cluster	Structure name	Number of voxels	Peak *t* value	*x*	*y*	*z*
1	Putamen_R, Caudate_R	65	5.1528	15	15	−6
2	Putamen_L	52	5.1872	−24	6	−3
3	Cingulum_Ant_L	37	5.3152	0	45	−3
4	Precuneus_R	54	4.7141	6	−45	9

Abbreviations: Caudate_R: right caudate nucleus; Cingulum_Ant_L: left anterior cingulate and paracingulate gyri; Precuneus_R: right precuneus; Putamen_L: left lenticular nucleus, putamen; Putamen_R: right lenticular nucleus, putamen.

**TABLE 3:** Brain regions with significant differences in mALFF between CKD and HC.

				Peak MNI Coordinates		
Cluster	Structure name	Number of voxels	Peak *t* value	*x*	*y*	*z*
1	Putamen_R, Caudate_R	273	5.6627	12	12	−6
2	Putamen_L, Caudate_L	191	5.808	−27	6	−3
3	Cingulum_Ant_L	72	5.2456	0	45	−3
4	Precuneus_R	147	4.6986	9	−45	9

Abbreviations: Caudate_L: left caudate nucleus; Caudate_R: right caudate nucleus; Cingulum_Ant_L: left anterior cingulate and paracingulate gyri; Precuneus_R: right precuneus; Putamen_L: left lenticular nucleus, putamen; Putamen_R: right lenticular nucleus, putamen.

### 3.3 Comparison of Regional Homogeneity in CKD and Healthy Control Groups

**FIGURE 3:**
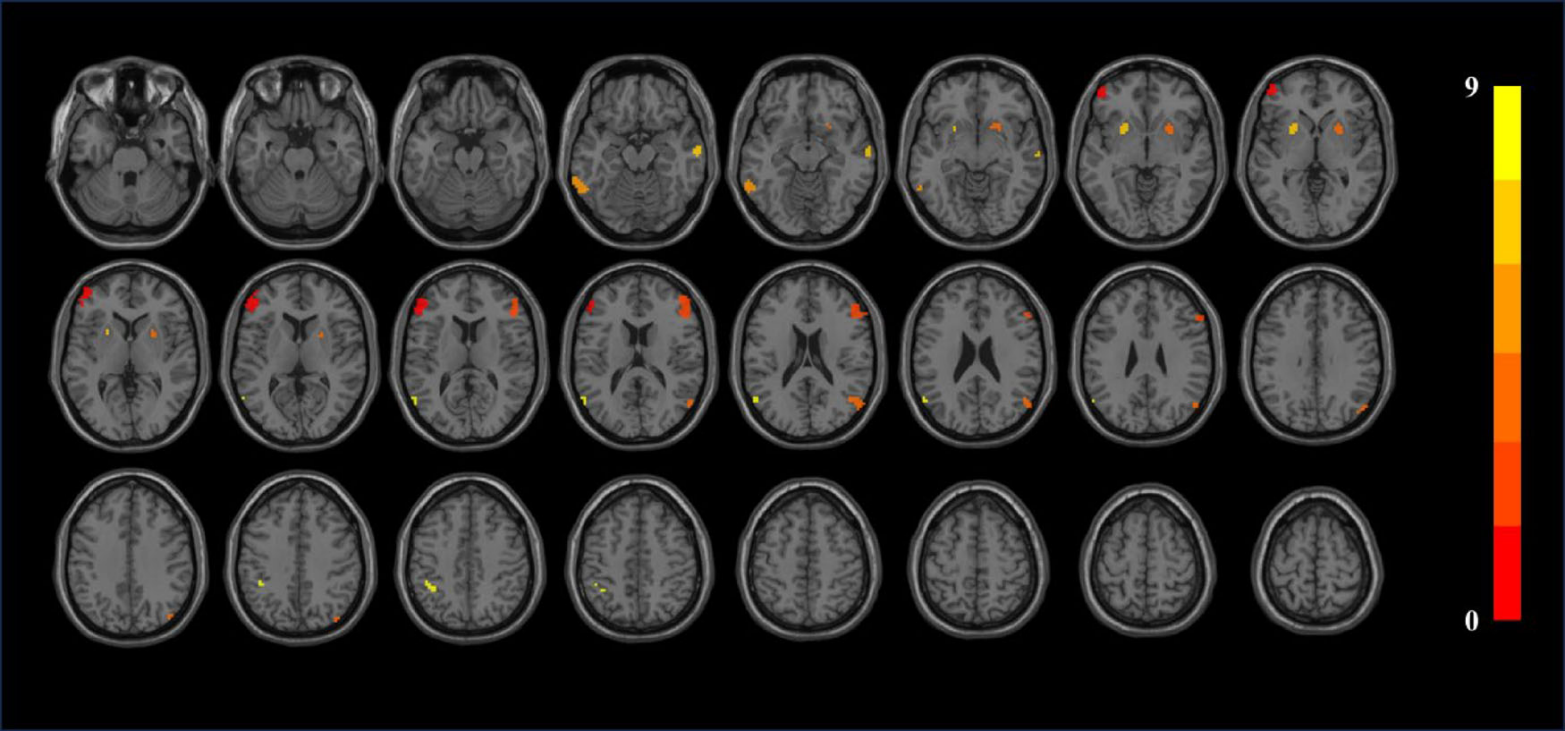
smCohe‐ReHo results. Difference between HC and CKD groups. The results were corrected by FDR, where *p *< 0.05 and cluster size over 20.

**FIGURE 4:**
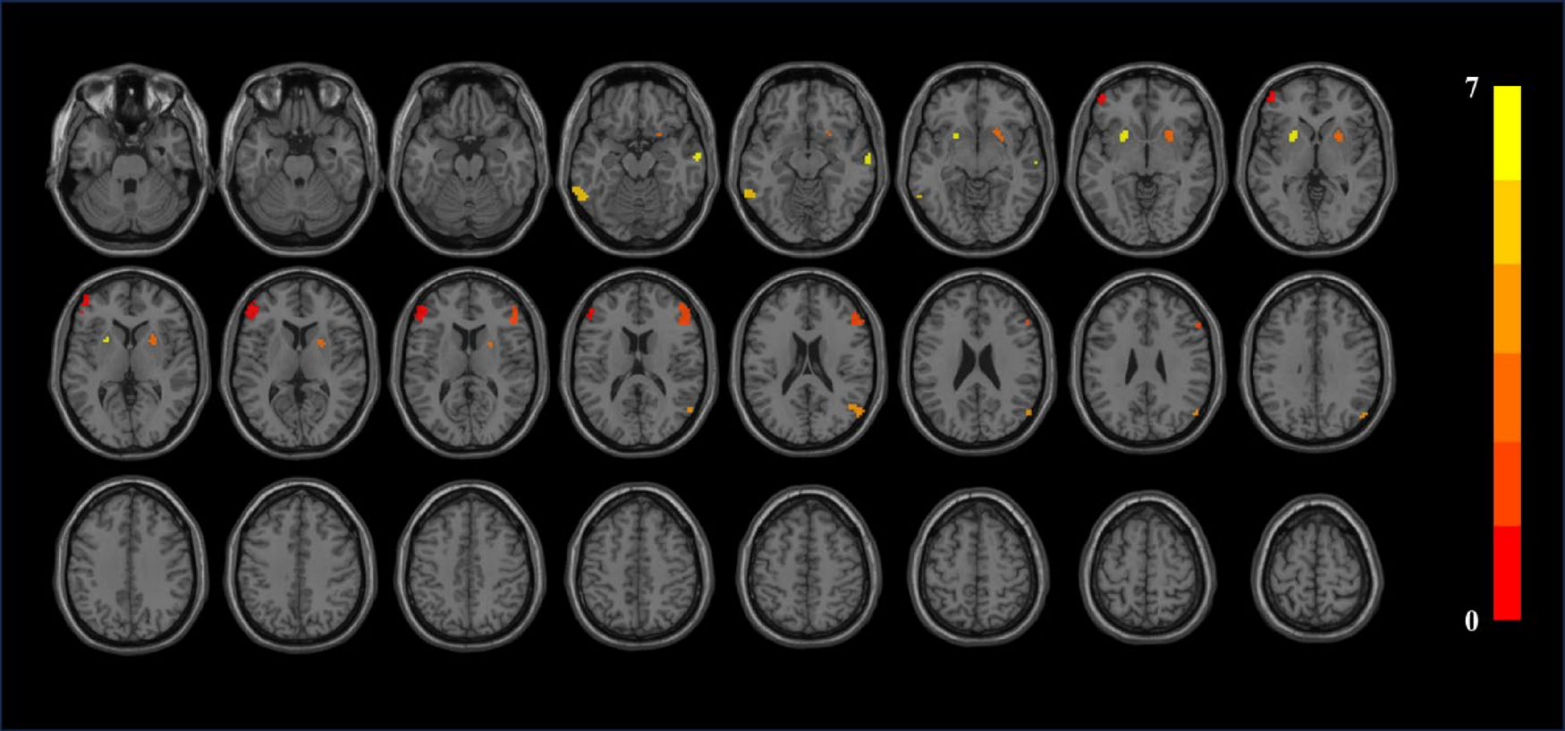
szCohe‐ReHo results. Difference between HC and CKD groups. The results were corrected by FDR, where *p *< 0.05 and cluster size over 20.

Table 4 and Figure 3 show the statistically significant changes of smCohe‐ReHo. Patients with CKD have decreased smCohe‐ReHo in the left inferior temporal gyrus, bilateral putamen, bilateral inferior frontal gyrus triangular part, bilateral middle frontal gyrus, right middle temporal gyrus, and right angular gyrus (*p *< 0.05, cluster size > 30). Table 5 and Figure 4 show the statistically significant changes of szCohe‐ReHo. Patients with CKD have decreased szCohe‐ReHo in the left inferior temporal gyrus, bilateral putamen, bilateral inferior frontal gyrus triangular part, right middle frontal gyrus, and right middle temporal gyrus (*p *< 0.05, cluster size > 30).

**TABLE 4:** Brain regions with significant differences in smCohe‐ReHo between CKD and HC.

				Peak MNI Coordinates		
Cluster	Structure name	Number of voxels	Peak *t* value	*x*	*y*	*z*
1	Temporal_Inf_L	49	5.1555	−63	−54	−15
2	Putamen_R	50	4.3034	24	12	−3
3	Putamen_L	34	5.1716	−24	12	−3
4	Frontal_Inf_Tri_L, Frontal_Mid_L	114	5.6831	−51	39	9
5	Frontal_Inf_Tri_R, Frontal_Mid_R	104	6.297	51	33	15
6	Temporal_Mid_R, Angular_R	63	5.1545	54	−69	30

Abbreviations: Angular_R: right angular gyrus; Frontal_Inf_Tri_L: left inferior frontal gyrus, triangular part; Frontal_Inf_Tri_R: right inferior frontal gyrus, triangular part; Frontal_Mid_L: left middle frontal gyrus; Frontal_Mid_R: right middle frontal gyrus; Putamen_L: left lenticular nucleus, putamen; Putamen_R: right lenticular nucleus, putamen; Temporal_Inf_L: left inferior temporal gyrus; Temporal_Mid_R: right middle temporal gyrus.

**TABLE 5:** Brain regions with significant differences in szCohe‐ReHo between CKD and HC.

				Peak MNI Coordinates		
Cluster	Structure name	Number of voxels	Peak *t*‐value	*x*	*y*	*z*
1	Temporal_Inf_L	41	5.0942	−63	−54	−15
2	Putamen_R	68	4.5052	24	12	−3
3	Putamen_L	37	5.3279	−24	12	−3
4	Frontal_Inf_Tri_L	98	5.6976	−51	39	9
5	Frontal_Inf_Tri_R, Frontal_Mid_R	91	6.387	51	33	18
6	Temporal_Mid_R	44	4.9204	54	−69	30

Abbreviations: Frontal_Inf_Tri_L: left inferior frontal gyrus, triangular part; Frontal_Inf_Tri_R: right inferior frontal gyrus, triangular part; Frontal_Mid_R: right middle frontal gyrus; Putamen_L: left lenticular nucleus, putamen; Putamen_R: right lenticular nucleus, putamen; Temporal_Inf_L: left inferior temporal gyrus; Temporal_Mid_R: right middle temporal gyrus.

### 3.4 Correlation of zALFF With Laboratory Indicators in CKD Patients

**FIGURE 5:**
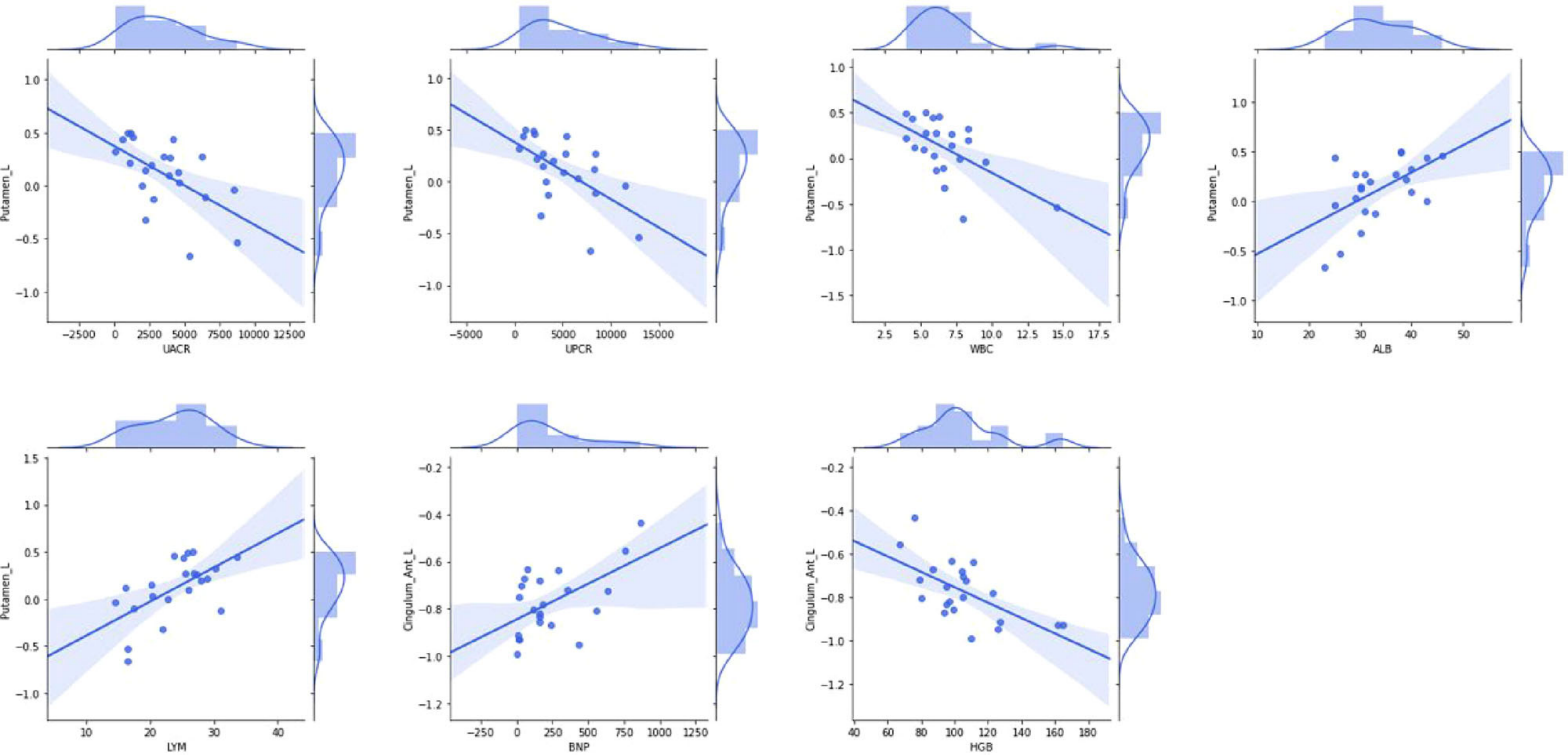
Correlation of zALFF with laboratory indicators (*R* ^2^ > 0.3, *p *< 0.05).

We selected the four clusters listed in Table 2 as ROIs, including the right putamen, left putamen, left anterior cingulate and paracingulate gyri, and right precuneus. Correlation analysis of zALFF and laboratory indicators is performed. The results are shown in Table 6 and Figure 5. No significant correlation is seen between zALFF and laboratory indicators in the right putamen and right precuneus. As the UACR (*R* ^2^ = 0.326, *p* = 0.005), UPCR (*R* ^2^ = 0.353, *p* = 0.004), and white blood cell (*R* ^2^ = 0.352, *p* = 0.004) levels increase, the zALFF values of the left putamen decrease. As the albumin (*R* ^2^ = 0.320, *p* = 0.006) and lymphocyte (*R* ^2^ = 0.367, *p* = 0.003) levels increase, the zALFF values of the left putamen increase. As the B‐type natriuretic peptide (*R* ^2^ = 0.312, *p* = 0.007) level increases, the zALFF values of the left anterior cingulate and paracingulate gyri increase. As the hemoglobin (*R* ^2^ = 0.393, *p* = 0.002) increases, the zALFF values of the left anterior cingulate and paracingulate gyri increase. No significant correlation is seen between zALFF and eGFR in any ROI. We plotted indicators with *R* ^2^ > 0.3 and *p* < 0.05 (Figure 5).

**TABLE 6:** Correlation analysis of zALFF and laboratory indicators.

	Putamen_R		Putamen_L		Cingulum_Ant_L		Precuneus_R	
	*R* ^2^	*p*‐value	*R* ^2^	*p*‐value	*R* ^2^	*p*‐value	*R* ^2^	*p*‐value
Age	0.024	0.487	0.022	0.508	0.025	0.485	0.262	0.015
BMI	0.012	0.629	0.003	0.796	0.121	0.113	0.137	0.090
eGFR (mL/min/1.73 m^2^)	0.077	0.212	0.032	0.426	0.051	0.314	0.039	0.376
HbA1C (%)	<0.001	0.952	0.003	0.794	0.016	0.576	< 0.001	0.990
BNP (pg/mL)	0.013	0.608	0.276	0.012	0.312	0.007	0.133	0.095
BUN (mmol/L)	0.104	0.143	0.005	0.757	0.010	0.654	< 0.001	0.989
SCr (µmol/L)	0.008	0.697	0.019	0.543	0.001	0.918	< 0.001	0.928
CO_2_CP (mmol/L)	0.011	0.641	0.001	0.886	< 0.001	0.938	0.036	0.397
ALB (g/L)	0.009	0.679	0.320	0.006	0.087	0.182	0.023	0.497
UP (g)	0.003	0.809	0.247	0.019	0.028	0.459	0.001	0.909
UACR (mg/g)	0.003	0.800	0.326	0.005	0.047	0.33	0.018	0.556
UPCR (mg/g)	0.006	0.726	0.353	0.004	0.043	0.354	0.027	0.465
CRP (mg/L)	0.040	0.37	0.001	0.869	0.009	0.679	0.006	0.729
ESR (mm/H)	0.017	0.561	0.022	0.508	0.284	0.011	0.138	0.088
PTH (pg/mL)	0.025	0.482	0.033	0.419	0.016	0.579	0.024	0.493
Ca2+ (mmol/L)	0.010	0.654	0.244	0.020	0.029	0.446	0.002	0.849
P (mmol/L)	0.081	0.199	0.016	0.579	0.038	0.387	< 0.001	0.948
WBC (*10^9^/L)	0.009	0.672	0.352	0.004	0.002	0.858	0.005	0.762
LYM%	0.012	0.634	0.367	0.003	0.056	0.290	0.075	0.218
HGB (g/L)	0.017	0.564	0.054	0.296	0.393	0.002	0.254	0.017

Abbreviations: ALB: albumin; BMI: body mass index; BNP: B‐type natriuretic peptide; BUN: blood urea nitrogen; Ca^2+^: calcium; Cingulum_Ant_L: left anterior cingulate and paracingulate gyri; CO_2_CP: carbon dioxide combining power; CRP: C‐reactive protein; eGFR: estimated glomerular filtration rate; ESR: erythrocyte sedimentation Rate; HbA1c: hemoglobin A1c; HGB: hemoglobin; LYM: lymphocyte; P: phosphorus; Precuneus_R: right precuneus; PTH: Parathyroid hormone; Putamen_L: left lenticular nucleus, putamen; Putamen_R: right lenticular nucleus, putamen; SCr: serum creatinine; UACR: urinary albumin creatinine ratio; UP: urine protein; UPCR: urinary protein creatinine ratio; WBC: white blood cell.

## 4 Discussion

We investigated the spatial patterns of intrinsic brain activity in patients with CKD using ALFF and ReHo. To mitigate the impact of related confounding factors, we employed a 1:1 matching method based on age and gender, ultimately including 22 CKD patients. We observed functional abnormalities in multiple brain regions of patients prior to dialysis. When compared to healthy controls, the zALFF, mALFF, smCohe‐ReHo, and szCohe‐ReHo values in the bilateral putamen exhibited statistically significant differences. Additionally, we found statistically significant correlations between the left putamen and several laboratory biomarkers reflecting CKD severity, including UACR, UPCR, and serum albumin.

Our study revealed a significant decline in zALFF and mALFF values in patients with CKD. ALFF is a measure of the intensity of spontaneous neural activity in the brain, which is believed to reflect the functional integrity of the brain (Yang et al. 2007). Both zALFF and mALFF are used in fMRI to standardize ALFF values across different subjects. Previous research has demonstrated that a decrease in ALFF values corresponds to reduced spontaneous brain activity (Markello et al. 2018). When compared to healthy controls, the CKD group exhibited decreased zALFF and mALFF values in several brain regions, including the bilateral putamen, right caudate nucleus, and right precuneus. Notably, a 2023 MRI study also reported decreased cerebral blood flow in the bilateral putamen, bilateral caudate, and right precuneus in non‐dialysis CKD patients (Wang et al. 2023). These brain regions overlapped with the regions showing statistical differences in ALFF values in our study. These findings collectively highlight the involvement of these brain regions in CKD‐related alterations.

ReHo analysis reflects the synchronization of whole‐brain voxels in localized regions of brain activity. Its measurements are positively correlated with the coherence and centrality of regional brain activity. In our study, we conducted Cohe‐ReHo analysis. We consistently observed a decrease in the smCohe‐ReHo and szCohe‐ReHo signals within the bilateral putamen, which aligns with the findings from ALFF. The putamen, a crucial nucleus within the basal ganglia, plays a role in striatum formation and is associated with reinforcement learning and motor control (Vinas‐Guasch and Wu 2017). Our investigation revealed that both ALFF and ReHo values in patients with CKD exhibited abnormalities in the bilateral putamen. This finding suggests that the connections involving the putamen are altered within the brain network of CKD patients. A recent rs‐fMRI study involving 64 CKD patients and 43 healthy controls demonstrated decreased fALFF values in the putamen when compared to healthy controls, while the ReHo values of the right putamen were altered in CKD patients (Yu et al. 2022). Additionally, another study utilizing a different MRI method found that as CKD progresses, patients exhibit higher susceptibility values in the putamen nucleus (Wang et al. 2023). These consistent results collectively indicate that the intrinsic brain activity within the bilateral putamen is likely to be altered in CKD patients.

In our study, we identified a linear relationship between zALFF values and several biomarkers in patients with CKD. Notably, as UACR and UPCR increased, zALFF values decreased. Additionally, a decrease in plasma albumin levels was associated with lower zALFF values. These observations are consistent with the simultaneous occurrence of increased UACR, UPCR, and decreased serum albumin levels in CKD patients. Urinary protein levels, including albumin and other proteins, serve as essential biomarkers for assessing kidney function. Elevated urinary protein levels, known as albuminuria, can indicate kidney damage or dysfunction, potentially affecting the filtration of waste products and nutrients within the body. For evaluating urine protein, both UACR and UPCR play crucial roles. As urinary protein levels rise, serum albumin levels tend to decline, resulting in a negative correlation between urinary protein and serum albumin levels. UACR serves as a sensitive indicator of early kidney damage and plays a pivotal role in managing and monitoring CKD progression (House et al. 2019; Ku et al. 2023). These biomarkers aid in diagnosing kidney disease, predicting the risk of cardiovascular events and mortality, and guiding treatment decisions.

In our study, we observed a correlation between the left putamen and UACR. However, given that CKD is a chronic and systemic disease, we were intrigued by the inconsistency in findings between the right and left putamen. Drawing from previous literature, we note that in several other systemic disorders, the left putamen has indeed demonstrated greater sensitivity compared to the right putamen. For instance, in systemic lupus erythematosus (SLE), an autoimmune disease affecting multiple systems, an fMRI study revealed a correlation between complement C3 and the left putamen, whereas no such correlation was found with the right putamen (Yu et al. 2019). Similarly, another fMRI study comparing individuals exposed to hypoxic conditions at high altitudes with those in normal environments solely identified a correlation between hemoglobin concentration and the left putamen (X. Zhang et al. 2023). However, there remains a lack of definitive explanations for this phenomenon.

However, our study has certain limitations. Firstly, it is a cross‐sectional study, which may introduce bias due to its observational nature. Secondly, the relatively small sample size could limit the generalizability of our findings. Larger‐scale studies are necessary to validate and confirm these results.

## 5 Conclusion

Non–dialysis‐dependent CKD patients had changes in zALFF, mALFF, smCohe‐ReHo, and szCohe‐ReHo values in specific brain regions, especially bilateral putamen. UACR, UPCR, and serum albumin levels are associated with putamen activity attenuation in rs‐fMRI. These findings highlight the importance of investigating brain–kidney interactions in non–dialysis‐dependent CKD patients, providing a basis for further exploration of the effects of CKD on the central nervous system and its potential mechanisms.

## Author Contributions

**Yangjie Yu**: writing–original draft, writing–review and editing, validation, conceptualization, visualization. **Jun‐Peng Zhang**: conceptualization, formal analysis, writing–original draft, writing–review and editing. **Zhen Wang**: data curation, software. **Juan Li**: data curation. **Xu‐Yun Hua**: conceptualization, methodology, writing–original draft, writing–review and editing. **Junjie Pan**: conceptualization, methodology, funding acquisition, writing–review and editing, writing–original draft, project administration. **Rui Dong**: writing–review and editing, writing–original draft, resources; conceptualization, data curation, supervision.

## Ethics Statement

This controlled study of CKD was ratified by the ethics committee of Shanghai Changhai Hospital, Naval Medical University (No. CHEC2022‐159).

## Conflicts of Interest

The authors declare no conflicts of interest.

### Peer Review

The peer review history for this article is available at https://publons.com/publon/10.1002/brb3.70106

## Data Availability

The data that support the findings of this study are available on request from the corresponding author. The data are not publicly available due to privacy or ethical restrictions.
